# β_2_ Adrenergic Receptor Fluorescent Protein Fusions Traffic to the Plasma Membrane and Retain Functionality

**DOI:** 10.1371/journal.pone.0074941

**Published:** 2013-09-23

**Authors:** Jaclyn Bubnell, Patrick Pfister, Maria L. Sapar, Matthew E. Rogers, Paul Feinstein

**Affiliations:** 1 Department of Biological Sciences, Hunter College and The Graduate Center Biochemistry, Biology and Biopsychology and Behavioral Neuroscience Programs, CUNY, New York, New York, United States of America; 2 Corporate Research and Development, Firmenich Inc., Plainsboro, New Jersey, United States of America; Cleveland Clinic Lerner Research Institute, United States of America

## Abstract

Green fluorescent protein (GFP) has proven useful for the study of protein interactions and dynamics for the last twenty years. A variety of new fluorescent proteins have been developed that expand the use of available excitation spectra. We have undertaken an analysis of seven of the most useful fluorescent proteins (XFPs), Cerulean (and mCerulean3), Teal, GFP, Venus, mCherry and TagRFP657, as fusions to the archetypal G-protein coupled receptor, the β_2_ adrenergic receptor (β_2_AR). We have characterized these β_2_AR::XFP fusions in respect to membrane trafficking and G-protein activation. We noticed that in the mouse neural cell line, OP 6, that membrane bound β_2_AR::XFP fusions robustly localized in the filopodia identical to gap::XFP fusions. All β_2_AR::XFP fusions show responses indistinguishable from each other and the non-fused form after isoprenaline exposure. Our results provide a platform by which G-protein coupled receptors can be dissected for their functionality.

## Introduction

G-protein coupled receptors (GPCRs) represent the largest family of eukaryotic membrane proteins [1]. The GPCR superfamily is natively expressed in many cell types; 90% of GPCRs are expressed in the brain. They are activated by almost every known neurotransmitter, peptide, and chemokine [2]. Mutations to GPCRs are known to cause a cascade of neurological and neurodegenerative diseases [3,4]. Common to all GPCRs is their seven transmembrane structure: seven alpha helical transmembrane domains connected by intra and extracellular loops with an extracellular amino terminus and an intracellular carboxyl terminus. The intracellular regions regulate G-protein binding, while ligand-binding properties exist in the extracellular and transmembrane domains [5]. GPCR’s are the target of over 50% of therapeutic drugs, and a quarter of the top marketed drugs target GPCRs [6].

GPCRs function at the plasma membrane where they bind ligand and are subsequently regulated by ligand dependent endocytosis. In order to study behavior of GPCRs, it is necessary to visually track their subcellular localization throughout the process of ligand activation, internalization, recycling, and degradation [7].

The β_2_ adrenergic receptor (β_2_AR) has served as the archetype of GPCRs. It binds ligands such as catecholamines and isoprenaline, which in turn induce a signal transduction cascade, catalyzing the exchange of GDP for GTP in the alpha subunit of the heterotrimeric G-protein. This reaction results in the activation of adenylyl cyclase leading to an increase in the concentrations of cyclic AMP in the cell [8]. The distribution of receptor in the cell upon ligand activation and the process of receptor internalization and resensitization have been well characterized [9,10].

One of the most useful methods for studying β_2_AR trafficking, distribution, internalization, and resensitization has been through the use of green fluorescent protein (GFP) fusions [11,12,13]. The overexpression of β_2_AR::GFP in cell lines does not affect its cellular activities and outcomes are readily quantifiable.

β_2_AR::GFP fusions have provided a tool for studying its subcellular localization. In the absence of ligands, it is found on the plasma membrane of multiple cell types. After agonist exposure, β_2_AR::GFP internalization is easily visualized within the endosomal compartments. The possibility of GPCRs existing as quaternary structures has been studied through fusions with GFP and snap tags, but the increased availability of photostable fluorescent proteins may provide another reliable method [7]. The β_2_AR has also been commonly fused to two GFP derivatives, the yellow fluorescent protein (YFP) and the cyan fluorescent protein (CFP), however, both new fluorescent proteins and improved versions of these proteins now exist that make visualization more efficient [14,15,16]. The availability of a wide range of fluorescent proteins that span the visible spectrum of light could make it possible to simultaneously compare multiple β_2_AR mutants or multiple GPCRs more effectively.

The ability to monitor subcellular biochemical processes has been revolutionized by the availability of fluorescent proteins from multiple species capable of self-synthesizing chromophore [17]. Fluorescent proteins are commonly used as molecular tags in analyses of protein function, protein-protein interactions, subcellular trafficking, and degradation [7,18]. Ever since the discovery of GFP from *Aequorea* *victoriae* , the development of fluorescent proteins in a variety of excitation spectra have been a major goal in the field. The toolbox of fluorescent tags has been proven invaluable to protein studies [19].

While many fluorescent proteins have been developed, a handful have proven to be the most useful for their photostability, detectability, narrow emission spectra, and low cellular toxicity. GFP, Venus, Cerulean, Teal, mCherry, and TagRFP657 are some of the most useful fluorescent proteins for the above reasons and have proven especially useful for colocalization and Förster resonance energy transfer (FRET) studies [19].

As an *Aequorea* GFP variant, Venus is one of the easiest to detect of fluorescent proteins. It is one of the most useful probes in the yellow class and can be used as an acceptor for FRET analysis with cyan fluorescent protein donors. It is highly photostable and contains a mutation, which allows it to mature more quickly and efficiently than GFP at 37°C [20]. Venus has been previously fused to the β_2_AR; however, full functional analyses have not been compiled [21,22].

Cerulean, another *Aequorea* derivative, is a cyan fluorescent protein with higher photostability and detectability compared with eBFP [23]. mCerulean3 is an improved version of Cerulean, which decreases susceptibility to photo bleaching and increases brightness by 67% [24]. Cerulean was considered the best choice in the cyan fluorescent protein family until the development of Teal (mTFP1) from the *Clavularia* sp. coral [25]. Teal is green shifted, but more easily detectable with higher photostability and acid insensitivity when compared with Cerulean and mCerulean3. Teal is a better choice of the cyan fluorescent proteins for colocalization studies; it narrows the emission spectra from 60nm with other cyan fluorescent proteins to only 30 nm. This makes it a more suitable donor for FRET analysis with Venus as the acceptor [26].

Good red-emitting fluorescent proteins (RFPs) have been difficult to develop [27]. They’re quite useful in that they have longer excitation, which provides less photo toxicity and can probe deeper into biological tissue. One of the most successful of the monomeric RFPs is mCherry from the *Discosoma* sp. coral. Its brightness is 50% of GFP, which is high for RFPs. It is also the most photostable of the reds [28].

Even more difficult to develop then the RFPs have been the far-reds. Recently, the red fluorescent protein mKate from the sea anemone *Entacmaea* *quadricolor* was shifted far-red with 10 amino acid substitutions. This variant has been named TagRFP657. The creation of this far-red protein now allows the use of three reds at once (TagRFP, mKate, and TagRFP657) for colocalization studies [29]. Recently, a more easily detectable version of TagRFP657 with a single lysine substitution, T10K, has been identified and is hereafter named auburn fluorescent protein (AFP).

We have tested a set of seven structurally different fluorescent proteins (Cerulean, mCerulean3, Teal, GFP, Venus mCherry, and AFP) derived from four distinct organisms as fusions to the mouse β_2_AR. We have characterized these chimeric proteins with respect to plasma membrane trafficking and response to ligand in order to determine if these fusions affect GPCR behavior. Our data show that trafficking and function of the β_2_AR is not affected by fluorescent protein fusions, making them useful tools for GPCR analyses. Our goal is to increase the resources for studying GPCR trafficking in order to elucidate structure function information.

## Materials and Methods

### Plasmid Construction

To create the *in vitro* expression vector (D346) for the β_2_AR fusion constructs, the peGFP-N1 (ClonTech) was digested with EcoRI and NotI to remove part of the multiple cloning site and GFP and an AscI site was added with linkers for cloning of other fluorescent protein fusion constructs (L34: 5’ AATTCGGCGCGCCAAAAGC 3’; L35: 5’ GGCCGCTTTTGGCGCGCCG 3’). (Figure S1.) 

To create the gap fluorescent protein fusion constructs, each fluorescent protein was amplified by PCR with primers containing the gap sequence at the 5’ end and EcoRI sites at the 5’ and 3’ ends (U196 : 5’ CATTCAAATATGTATCCGCTCATGAGACAATAACCCTGATAAATGCTTCAATAATATTGAAAAAGGAAGAGTGCCACCATGCTGTGCTGCATCAGAAGAACTAAGCCGGTTGAGAAGAATGAAGAGGCCGATCAGGAGATGGTGAGCAAGGGCGAGG 3’ and P422: 5’ TTACTTGTACAGCTCGTCCATG 3’). The PCR product was shuttled into the pGEM-T Easy (Promega) vector system. Each pGEM-T/gap fluorescent protein construct was then digested with EcoRI and shuttled into D346 (Figure S1 B).

To create the β_2_AR fusion constructs, the mouse (129) β_2_AR coding sequence was amplified by PCR to remove the stop codon and to add a 5’ AscI site and a *3’* PacI site (File S1 for sequences). The PCR product was shuttled into the pGEM-T Easy Vector system (Promega) followed by a digest with EcoRI (from pGEM-T)*/*PacI and shuttled into a modified D346 vector. The D346 vector already contained a GPCR fusion protein scaffold as follows: EcoRI*-*AscI-GPCR-*PacI*-::GFP-*AscI-NotI*. This created the parent construct D357, β_2_AR::GFP for all other XFP insertions. The other six fluorescent proteins were amplified by PCR with primers P463 (5’ AAAGTCTTTTTAATTAACGATCCACCGGTCGCCACCATGGTGAGCAAGGGCGAGGAG 3’) to add a 5’ end PacI site and P196: (5’ TTTTGCGGCCGCTTACTTGTACAGCTCGTCCATGC 3’) to add a 3’ end NotI site or P422 (5’ TTACTTGTACAGCTCGTCCATG 3’) using the NotI site from pGEM-T. The β_2_AR /peGFP-N1 construct and the fluorescent protein/pGEM-T constructs were doubled digested with *PacI/NotI* and each fluorescent protein was shuttled into the β_2_AR /peGFP-N1 parent construct (Figure S1 C). TagRFP657 was modified with a T10K mutation and GFP ends, as found in addgene, and confirmed by Vladislav Verkhusha as being more stable (personal communication). In our analyses, TagRFP657 was virtually undetectable as compared to AFP. To create the untagged fluorescent protein constructs, each fluorescent protein (P463/P196 or P422) was digested with EcoRI or *EcoRI/NotI* out of pGEM-T and shuttled into the *in vitro* expression vector (Figure S1 A). Templates for mCerluean3 and TagRFP657 were derived from minigenes (Genewiz). See File S1 for sequences.

### Culture and Transfection of OP6 Cells

Mouse olfactory placode cells (OP 6 [30], a gift from Jane Roskams) were maintained in Dulbecco’s modified Eagle’s medium (DMEM 1X Gibco) supplemented with 10% fetal bovine serum (FBS, Gibco) and 1% penicillin/streptomycin (Pen Strep, Millipore) at 33°C.

Plasmid DNA constructs were transiently transfected using the Amaxa Nucleofector (Lonza) with PBS at 60%-70% confluency according to the manufacturer’s protocol. Transfected cells were allowed to recover for 24 hours at 33°C and express the plasmid DNA.

For ligand activation experiments, OP 6 cells were exposed to 10 µM of isoprenaline (Sigma Aldrich Cat. # 15627) at 33°C. Live imaging of OP 6 cells was performed in culture media, except for labeling of the early endosomal compartments, which was performed in Opti-MEM (Gibco, Cat. #10569) with 20µg/ml of Alexa Fluor labeled transferrin (488 or 647, Life Technologies Cat. #s T-13342, T-23366) for 30 minutes before imaging.

### Immunostaining

After co-transfection with β2AR::XFP and a myc-tagged β-arrestin2 (OriGene), OP 6 cells were washed once with PBS (Millipore) and either exposed to 10 µM of isoprenaline for 20 minutes and then fixed in 10% formalin (Fisher Scientific) for 20 minutes at room temperature (RT), or fixed immediately without stimulation of isoprenaline. Cells were then washed three times with RT PBS and blocked in blocking buffer containing 0.3% Triton X-100 (Roche) and 5% horse serum (Gibco) in PBS for 1h at RT. Subsequently, an anti-myc (rabbit) polyclonal antibody (Santa Cruz Cat. # sc-789) was added to the cells in a 1:100 dilution and incubated overnight at 4°C. Cells were then washed three times with PBST buffer (0.3% Triton X-100 in PBS), and blocking buffer with 1: 500 goat anti-rabbit Alexa Fluor 546 or 488 (Life Technologies) was added for 1 h at RT. The cells were then imaged in PBS immediately after immunostaining.

For detection of wild type β_2_AR, antibody against β_2_AR (goat; Santa Cruz Cat# sc-570) was used at a 1:100 dilution followed by a 1:500 dilution of goat anti-rabbit Alexa Fluor 488 secondary antibody.

### Laser Scanning Confocal Microscopy

OP 6 cells were imaged on a Zeiss LSM 510 microscope using a Zeiss, Plan-APO 25X water immersion objective. Cerulean, mCerulean3, and Teal were excited at 458 nm and collected at BP465-510 nm, GFP was excited at 488 nm and collected at BP500-545 nm. Venus was excited at 514 nm collected at LP520 nm, mCherry was excited at 561 nm and collected at LP575 nm, and AFP was excited at 633 nm collected at LP650 nm. All images were acquired using the multi-tracking feature. The time-lapse for β_2_AR::AFP was done on the LSM510 nm using excitation at 630 nm and collected at LP650 nm.

### Spinning Disk Confocal Microscopy

For time-lapse experiments, OP 6 cells expressing the β_2_AR fusion constructs were exposed to 10µM isoprenaline and single plane images were taken every 5 minutes for 20 minutes on one of two spinning disk microscopes. For β_2_AR::GFP, β_2_AR::mCherry, and β_2_AR::Venus, an inverted LEICA CTR4000 microscope with Hamamatsu EM-CCD camera and CSU10 Yokogawa confocal unit was used with a LEICA 1.4 NA 60x oil immersion lens. β_2_AR::GFP and β_2_AR::Venus were excited at 491 nm and collected at BP(FF01-520/35 nm). β_2_AR::mCherry was excited at 561 nm and collected at BP620/660 nm. Images were obtained using the Volocity software.

β_2_AR::Cerulean, β_2_AR::mCerulean3, and β_2_AR::Teal were imaged using the PerkinElmer ultraVIEW ERS system, which includes an inverted Nikon ECLIPSE TE2000-U microscope with Hamamatsu ORCA-EM camera and PerkinElmer ultraVIEW PhotoKinesis confocal unit, and a LEICA 1.4 NA 60x oil immersion lens. β_2_AR::Cerulean, β_2_AR::mCerulean3, and β_2_AR::Teal were excited at 440 nm and collected at LP485 nm. Images were obtained using the Volocity software.

### Dose Response

For the dose response assay experiments, 1 x 10^4^ HEK293T cells were seeded in a 96 well assay plate (Corning – Costar, Cat. # 3842), a black, clear bottom, poly-D-Lysine coated plate for Fluorometric Imaging Plate Reader (FLIPR) screening. Cells were grown in cell culture media DMEM with 10% FCS without antibiotics until the next day. Transfections were performed on a per well basis according to a modified Lipofectamine 2000 reagent protocol (Invitrogen, Cat. # 11668-019). 42ng of both the receptor DNA and the human Galpha15 DNA were pooled in 4.2µl of Opti-MEM (Gibco, Cat. #31985). 29nl of Lipofectamine 2000 was added in 4.2µl of Opti-MEM. After five minutes of incubation at room temperature, solutions were mixed and left at RT for another 15min. 42µl of culture media was added and the final solution added to the cells. Transfection media was removed after 24 hours, and cells were allowed to recover for six hours in culture media before the FLIPR assay.

### Statistical tests

To determine the significance of filopodia in cells expressing β_2_AR::XFPs or gap::XFPs in comparison to untagged fluorescent proteins, a two-tailed Fishers exact test using the method of summing small P values was performed for each fluorescent protein using the online Graph Pad calculator. This test was also used to determine the significance of the increase in Intensity Correlation Quotient (ICQ) values in colocalization experiments.

For transferrin and β-Arrestin2 experiments, images were processed in ImageJ for Intensity Colocalization Analysis (ICA) as described by Li et al [31]. The protocol for ICA in ImageJ was obtained from the online manual for the WCIF-ImageJ collection through the Wright Cell Imaging Facility (http://www.uhnresearch.ca/facilities/wcif/imagej/colour_analysis.htm). The Intensity Correlation Quotient (ICQ) values were obtained for cells prior to isoprenaline exposure and after isoprenaline exposure. Colocalization analysis for each β_2_AR::XFP combined visualization of fluorescent markers with change in ICQ values after isoprenaline stimulation. 

## Results

### Localization of Untagged Fluorescent Proteins (XFPs)

Do our set of untagged fluorescent proteins contain any internal signals for trafficking to the plasma membrane? We developed a transient transfection system using an olfactory placode cell line (OP 6) to test the fluorescent proteins expressed under a CMV promoter (Figure S1 A). OP 6 cells exhibit several morphological features common to neurons including long axonal like extensions and numerous filopodia. After 24 hours, each fluorescent protein was diffusely distributed in the cytoplasm (Figure S2 A-G, Ai-Gi). GFP, Venus, mCherry, and Teal were the best detected with the highest stability while Cerulean, mCerulean3, and AFP were more difficult to detect and sustained higher instances of photobleaching.

### Plasma Membrane Expression of gap::XFPs and β_2_AR::XFPs

**Figure 1:**
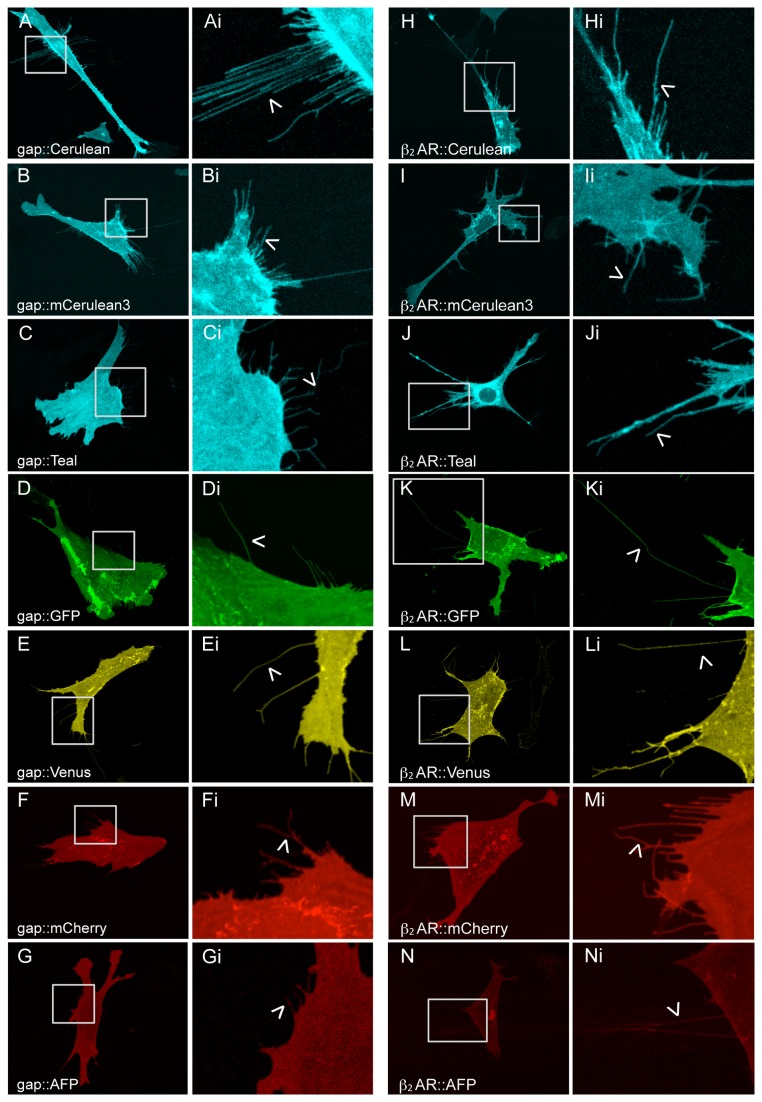
OP 6 cells transiently expressing gap::XFPs and β_2_AR::XFPs. Single cells expressing each gap tagged fluorescent protein localizes to the plasma membrane (A-G). gap::XFPs also target to the filopodia (arrowheads, Ai-Gi magnified images). Single cells expressing each β_2_AR fusion (β_2_AR::XFP) (H-N) exhibit diffuse protein expression at the plasma membrane defined by localization at the filopodia (arrowheads, Hi-Ni magnified images). The ability of β_2_AR to traffic to the plasma membrane is unaffected by fusion to the seven tested fluorescent proteins.

To define plasma membrane expression of each fluorescent protein in OP 6 cells, a 20 amino acid sequence (gap) from Zebrafish gap43 protein containing an amino terminal palmitoylation site was fused to Cerulean, mCerulean3, Teal, GFP, Venus, mCherry, and AFP [32,33]. The gap fusion constructs were transiently transfected into OP 6 cells and imaged after 24 hours. All gap fusions (gap::XFPs) were diffusely distributed at the plasma membrane and localized to the filopodia of the cell (Figure 1 A-G). OP 6 cells have a distinct morphology and display long filopodia, which are highly membranous (Figure 1 Ai-Gi). The fluorescence of Venus, GFP, mCherry, and Teal again were easiest to detect, while Cerulean, mCerulean3, and AFP were harder to detect and prone to photobleaching, respectively. Since expression of protein in the filopodia was unique to the gap::XFP constructs, this feature of OP 6 cells was used to define plasma membrane expression. Of note, the fluorescence of untagged XFPs appeared ill defined compared to the discreet gap::XFPs fluorescence in the OP 6 cells.

To determine if GPCR fusions to Cerulean, mCerulean3, Teal, GFP, Venus, mCherry, and AFP affect trafficking and ligand binding, constructs were created in which each fluorescent protein is fused to the carboxyl terminus of the 2 adrenergic receptor (β_2_AR). At a minimum, the β_2_AR::XFP fusions should localize to the plasma membrane where the β_2_AR normally functions. To determine if each β_2_AR fluorescent protein fusion can traffic to the plasma membrane, OP 6 cells were transfected with each β_2_AR fusion and assayed for localization (Figure 1 H-N). All cells expressing the β_2_AR fluorescent protein fusions showed fluorescence indistinguishable from gap::XFPs including localization to the filopodia (Figure 1 Hi-Ni). Like the gap::XFP fusions, Venus was the easiest to detect with highest photostability followed by GFP, mCherry, Teal, mCerulean3, Cerulean, and AFP.

### Filopodia Counts for Untagged Fluorescent Proteins, gap::XFPs, and β_2_AR::XFPs

**Figure 2:**
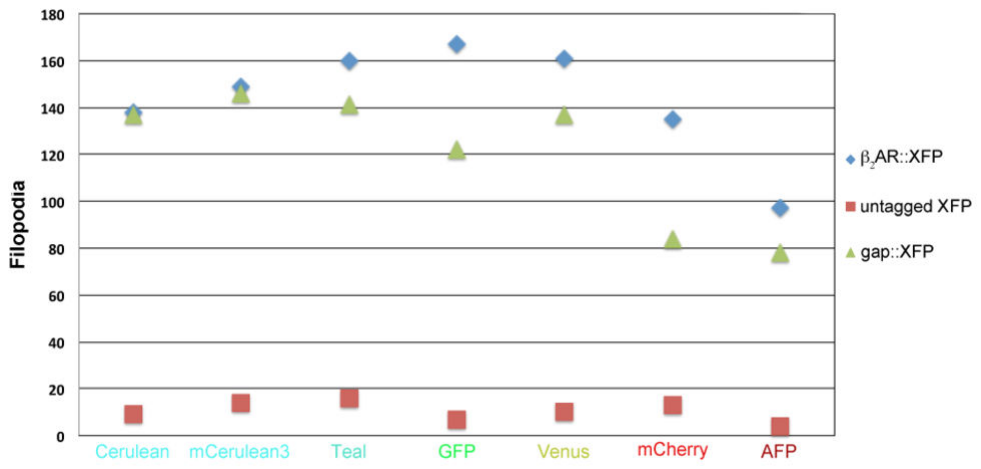
Filopodia count in OP 6 cells transiently expressing untagged fluorescent proteins, gap::XFPs, or β_2_AR::XFPs. Filopodia on N=10 cells were counted for cells expressing each untagged fluorescent protein, tagged with gap, or fused to the β_2_AR. gap::XFPs featured 78-141 filopodia per 10 cells, and β_2_AR::XFPs featured 97-167 filopodia per 10 cells. Cells expressing the untagged fluorescent proteins featured between 7-16 filopodia. Expression in filopodia of gap::XFPs and β_2_AR::XFPs was significantly different from the untagged XFPs, Fisher’s exact test P<0.0001.

To determine if protein localization at the filopodia was a means to identify plasma membrane expression in cells, each untagged fluorescent protein, gap::XFP, and β_2_AR::XFP were expressed, and filopodia of 10 random cells were counted. The total filopodia counts for gap::XFPs were 78-141; β_2_AR::XFPs were 97-167; as compared to untagged fluorescent protein were 4-16 (Figure 2). The numbers of filopodia present in β_2_AR::XFPs and gap::XFPs were indistinguishable and both were significantly different from untagged XFPs (Fishers exact test P<0.0001).

### Internalization of β_2_AR::XFPs

Membrane trafficking is only one measure of the functionality of a GPCR. Internalization of the β_2_AR::XFPs was investigated after acute stimulation by isoprenaline in OP 6 cells. Prior to stimulation, β_2_AR::XFPs are found in the plasma membrane with some perinuclear punctate expression, most likely due to over expression of the protein construct. After stimulation with 10µM isoprenaline, rapid internalization of the β_2_AR fusions into a punctate pattern was observed. Both the size and number of punctate aggregations of the β_2_AR fusions increase until the end of the 20-minute time course movies (Movies S1–S8). This pattern was obvious for all β_2_AR::XFP constructs, indicating that there is no adverse affect to internalization experienced by the fusion of the seven tested fluorescent proteins to the β_2_AR. By contrast, in the absence of ligand β_2_AR::GFP localization was unchanged after a 20-minute time course (Movie S8). Internalization was mediated by the β_2_AR::XFP fusions whereas gap::Teal coexpressed with untagged Venus was not internalized by isoprenaline and no punctate intracellular expression was observed (Figure S3 A, 0 and 20-minutes). OP 6 cells do not express β_2_AR (Figure S3 Bi-iii, C), ensuring internalization of cell surface proteins was not a result of endogenous expression. However, it was confirmed that OP 6 cells express the Gs_α_ or Golf_α_ G-protein subunit (data not shown).

### Colocalization of β_2_AR::XFP with Transferrin after Ligand Stimulation

**Figure 3:**
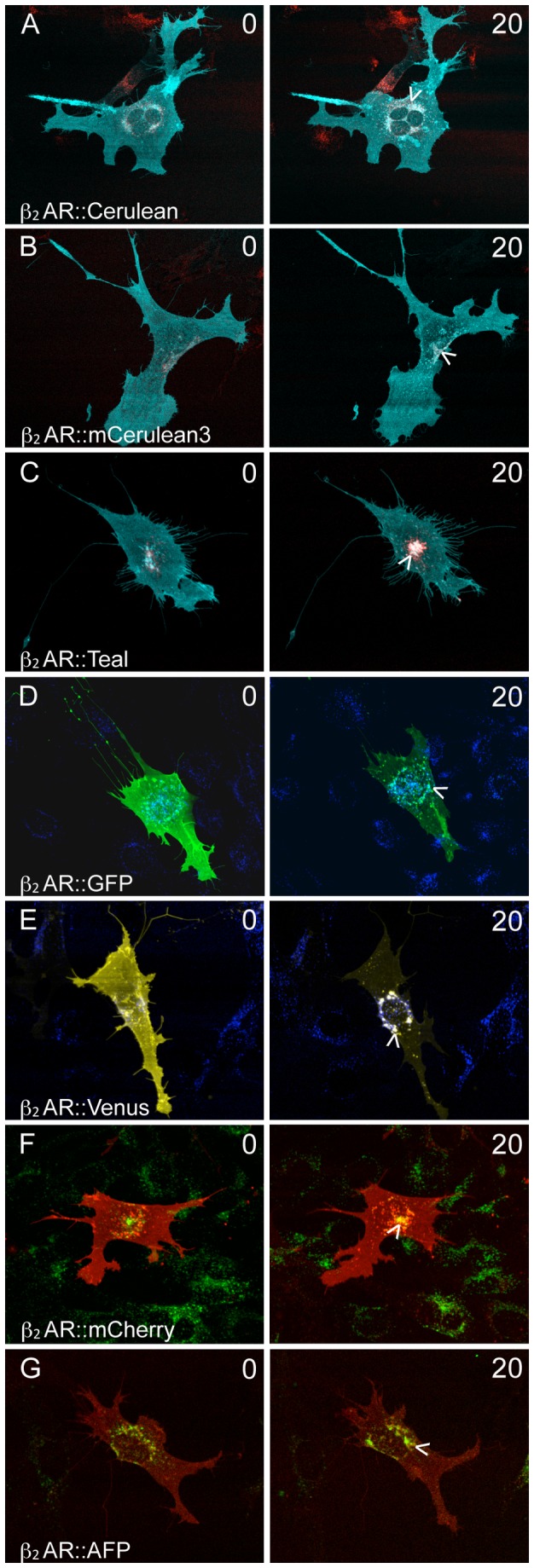
Colocalization of β_2_AR::XFP and transferrin after isoprenaline exposure. Single OP 6 cells expressing each β_2_AR::XFP construct were incubated with 20µg/ml of Alexa Fluor transferrin 647 (A-E) or Alexa Fluor transferrin 488 (F, G) for 30 minutes and then exposed to 10µM isoprenaline for 20 minutes. β_2_AR::XFP is diffusely expressed at the plasma membrane, and transferrin is localized in the endosomes (A-G, 0) before isoprenaline exposure. After 20 minutes, β_2_AR::XFP is internalized and becomes punctate, colocalizing with transferrin (arrowheads). ICQ values for all fusions reflect a significant increase (P<0.0001) in dependency after exposure to isoprenaline. ICQ values for 0 minute and 20 minute isoprenaline stimulation: (A) 0.086, 0.186 (B) 0.046, 0.187 (C) 0.116, 0.227 (D) -0.006, 0.212 (E) 0.132, 0.319 (F) 0.013, 0.254 (G) 0.047, 0.133.

A second measure of β_2_AR::XFPs functionality after stimulation by ligand is its uptake into early endosomes for recycling. The pathway for transferrin endocytosis is known to be identical to that of the β_2_AR. Live cells were incubated with Alexa Fluor 647 labeled transferrin or Alexa Fluor 488 labeled transferrin for 30 minutes, followed by stimulation with 10µM isoprenaline for 20 minutes. Single cells were imaged prior to the addition of isoprenaline, and again after 20 minutes of isoprenaline exposure. Prior to ligand stimulation, β_2_AR::XFPs are diffusely distributed at the plasma membrane, and transferrin is punctate and localized perinuclearly with little colocalization with any of the β_2_AR fluorescent protein fusions as shown by low dependence ICQ values (Figure 3 A-G, 0 minutes). After stimulation with isoprenaline for 20 minutes, each fusion becomes punctate and colocalizes with transferrin (Figure 3 A-G, 20 minutes). The intensity correlation quotients (ICQ) revealed that all β_2_AR::XFPs colocalized with transferrin (P<0.0001; see figure legend for ICQ values).

### Colocalization of β_2_AR::XFPs with β-Arrestin2 after Ligand Stimulation

**Figure 4:**
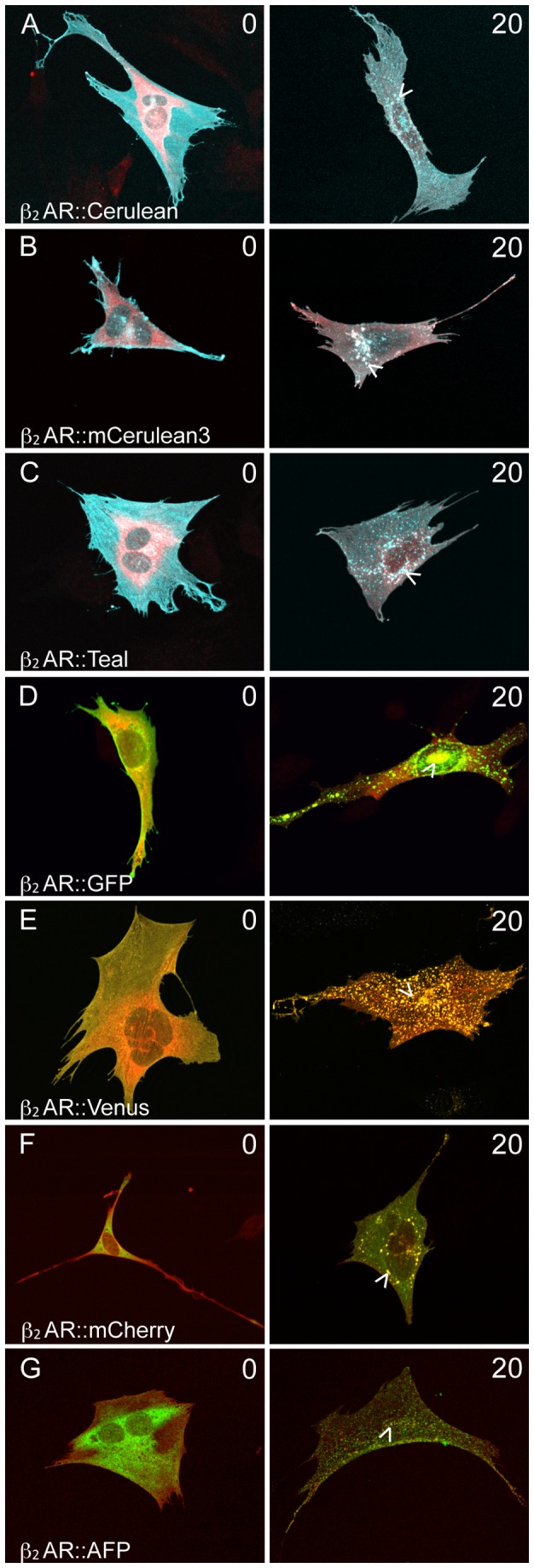
Colocalization of β_2_AR::XFP and β-Arrestin2 after isoprenaline exposure. Single OP 6 cells expressing β_2_AR::XFP and myc-tagged β-Arrestin2 were fixed after 24 hours and incubated with rabbit polyclonal antibody for Myc in the absence of isoprenaline, or after 20 minutes of exposure to 10µM isoprenaline. Anti-rabbit Alexa Fluor 546 was used for A-E, and anti-rabbit Alexa Fluor 488 was used for F, G. In the absence of isoprenaline (0 minutes), β_2_AR::XFP exhibits diffuse expression at the plasma membrane (A-G). After 20 minutes of exposure to 10µM isoprenaline, β_2_AR::XFP becomes punctate and colocalizes with β-Arrestin2 (arrowheads). ICQ values for all fusions reflect a significant increase (P<0.0001) in dependency after isoprenaline exposure. ICQ values for 0 minute and 20 minute isoprenaline stimulation: (A) 0.045, 0.107 (B) 0.098, 0.375 (C) 0.01, 0.17 (D) 0.136, 0.325 (E) 0.199, 0.381 (F) 0.014, 0.215 (G) -0.014, 0.139.

β-Arrestin2 is a regulatory protein involved in desensitization and sequestration of β_2_AR after agonist binding and phosphorylation [34]. After ligand stimulation, it is necessary for β_2_AR::XFPs to be regulated by β-Arrestin2 for proper functionality. β-Arrestin2 was not detectable in OP 6 cells by immunostaining (data not shown), so cells were cotransfected with a β_2_AR fusion and a myc-tagged β-Arrestin2 construct. In the absence of isoprenaline, all β_2_AR::XFPs were diffusely distributed at the plasma membrane, and β-Arrestin2 (detected by a polyclonal myc antibody) was diffusely distributed in the cytoplasm with little colocalization with the β_2_AR fusions as shown by low dependence ICQ values (Figure 4 A-G, 0 minutes). After exposure to isoprenaline, all β_2_AR fusions colocalized with β-Arrestin2 with high dependence described by ICQ values (Figure 4 A-G, 20 minutes; see figure legend for ICQ values). The internalization was marked by punctate perinuclear expression with some expression remaining at the plasma membrane.

### Time Course Colocalization Profiles of β_2_AR::Teal and β_2_AR::Venus

**Figure 5:**
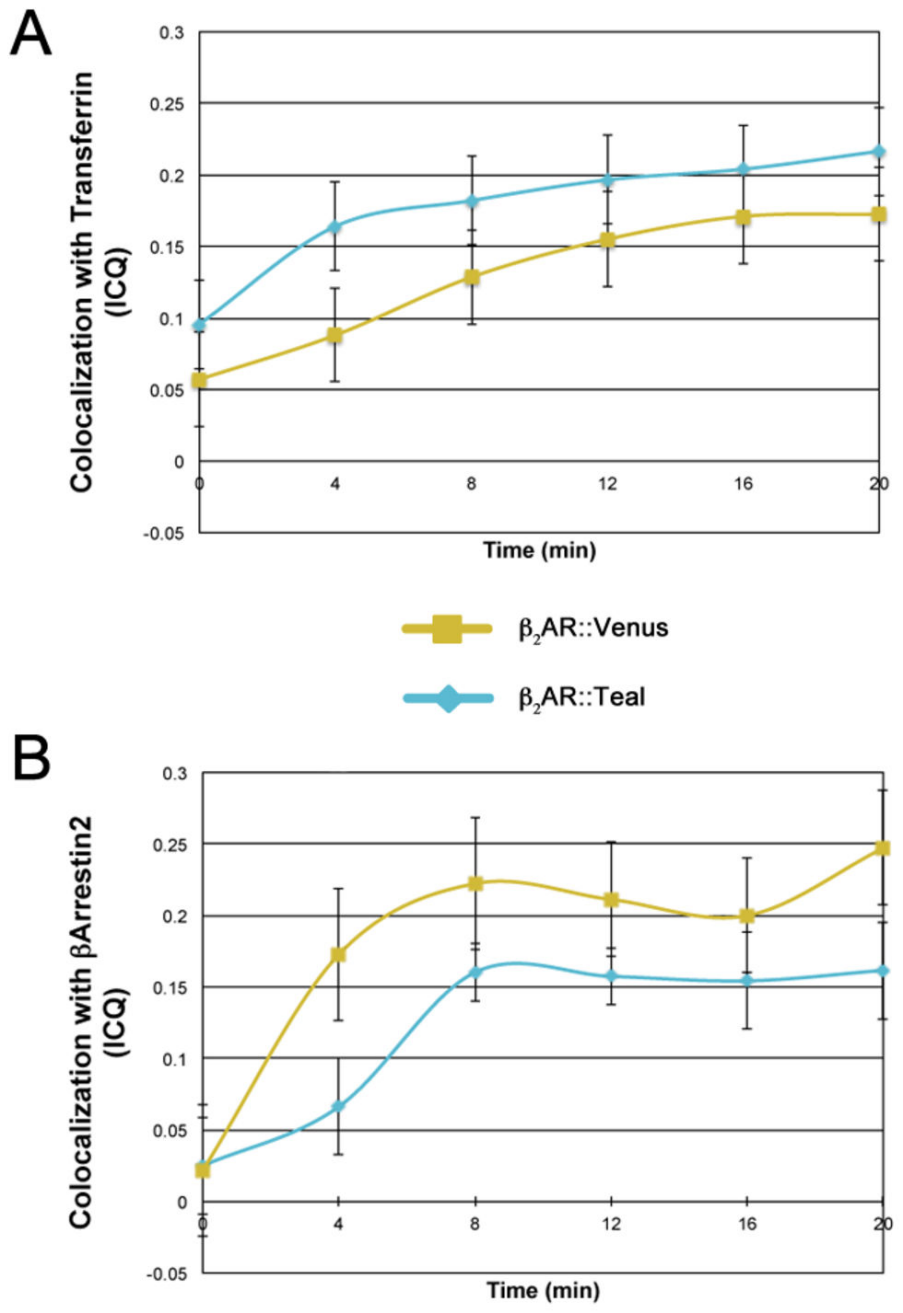
Detailed time course analysis of β_2_AR::Teal and β_2_AR::Venus. ICQ values were used to measure colocalization events. (A) Single OP 6 cells (n=2) expressing the β_2_AR::Teal or β_2_AR::Venus construct were incubated with 20µg/ml of Alexa Fluor transferrin 647 for 30 minutes and then exposed to 10µM isoprenaline and imaged live every 4 minutes until 20 minutes. (B) Single OP 6 cells (n=3,4) expressing the β_2_AR::Teal or β_2_AR::Venus construct and myc-tagged β-Arrestin2 were fixed after 24 hours and incubated with rabbit polyclonal antibody for Myc after exposure to 10µM isoprenaline in 4 minute intervals between 0 and 20 minutes and were visualized with Anti-rabbit Alexa Fluor 546 antibody. Fusions of Teal and Venus to the β_2_AR follow the same time course for internalization into the early endosomes (A) and the same time course regulation by β-Arrestin2 (B). Error bars represent standard error.

Teal and Venus have been previously characterized as excellent pairs for FRET analysis. To assure these fluorescent proteins did not affect the time course of β_2_AR internalization and could therefore be used as a FRET pair with GPCRS, colocalization of β_2_AR::Teal and β_2_AR::Venus constructs with transferrin and β-Arrestin2 after isoprenaline exposure were determined every 4 minutes for a total of 20 minutes using ICQ values (Figure 5 A,B). Both β_2_AR::Teal and β_2_AR::Venus were steadily internalized into the early endosomes as shown by corresponding increases in ICQ values over the 20 minute time course (Figure 5 A). Both β_2_AR::Teal and β_2_AR::Venus were able to interact indistinguishably with β-Arrestin2 showing sharp increases in dependency (ICQ) with β-Arrestin2 that plateau by the 8 minute time point (Figure 5B).

### Dose Response Profiles of β_2_AR::XFPs to Isoprenaline

**Figure 6:**
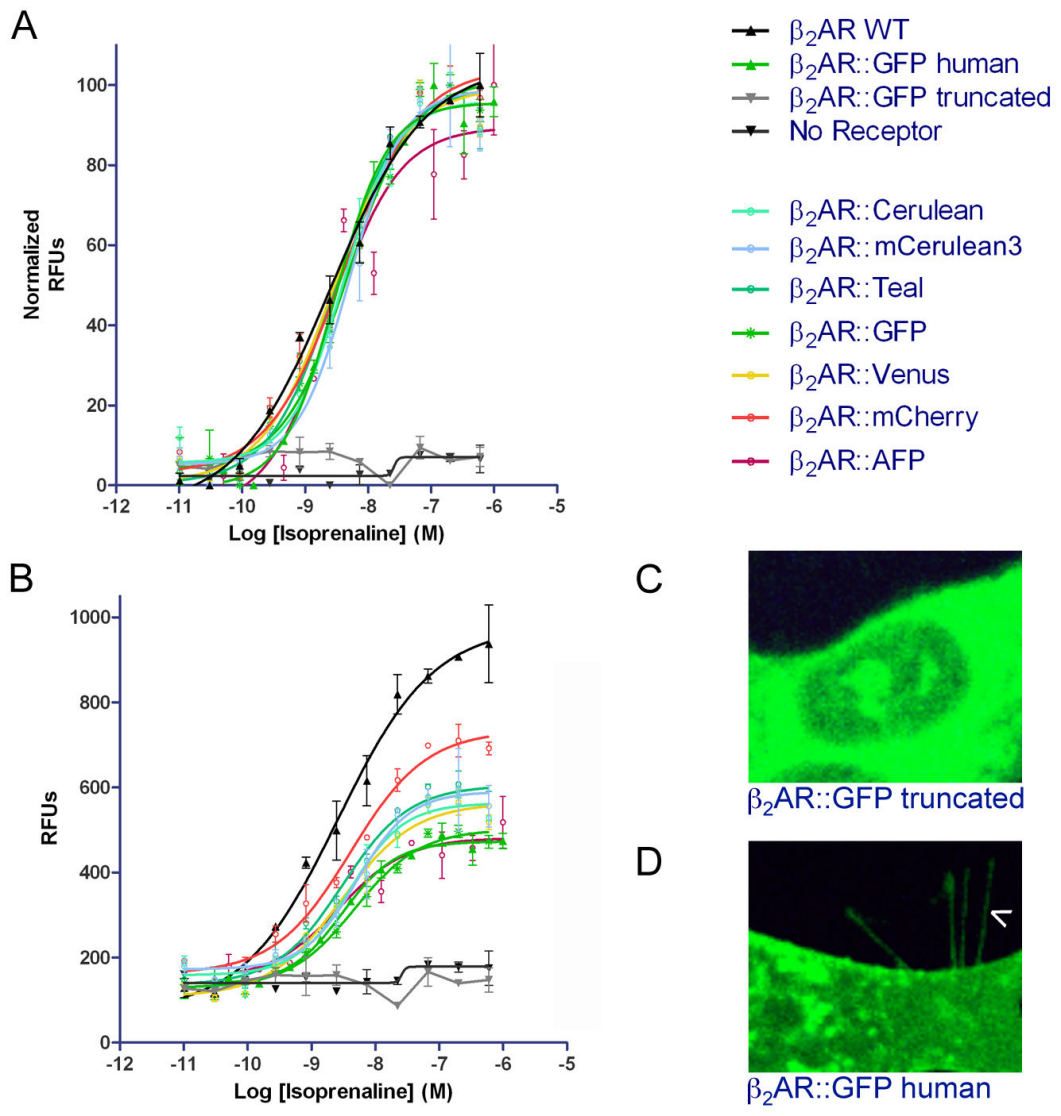
Dose response curves for β_2_AR::XFPs. Cells expressing each β_2_AR::XFP and human Gα15 were exposed to concentrations of isoprenaline and analyzed using the FLIPR assay. Normalized curves (A) show an EC50 between 2.6-5.2 x 10^-9^ for all fusions. Unnormalized curves (B) show a difference in maximum RFUs for individual fluorophore fusions. Transient expression of β_2_AR::GFP truncated reveals no membrane expression (C) whereas β_2_AR::GFP human localizes to filopodia (D, arrowhead).

Full functionality was finally assessed by the degree to which β_2_AR::XFPs were able to activate G-proteins. The FLIPR calcium assay was used to measure the release of internal calcium stores mediated by the Gq pathway (i.e. Gα15) in response to isoprenaline exposure. Response to increasing concentrations of ligand by β_2_AR::XFPs and subsequent human Gα15 activation was measured in relative fluorescent units (RFUs). Normalized dose response curves for all β_2_AR::XFPs had virtually identical EC50’s, ranging from 2.6-5.2 x 10^-9^ M similar to the unfused mouse β_2_AR and to the human β_2_AR::GFP, which had EC50s of 2.6 x 10^-9^ and 3.1 x 10^-9^, respectively (Figure 6 A, Figure S4). Unnormalized dose response curves for each fluorescent protein fusion compared to unfused mouse β_2_AR revealed a decrease in maximum RFUs (Figure 6 B, Figure S4). β_2_AR::GFP with a 90 amino acid carboxyl terminal truncation to arginine 328 (β_2_AR::GFP truncated), did not traffic to the plasma membrane despite containing all seven transmembrane domains, and did not show a response to ligand (Figure 6 A-C). β_2_AR::GFP truncated localized perinuclearly and was most likely retained in the endoplasmic reticulum. This is in contrast to human β_2_AR::GFP, which did traffic to the filopodia and was stimulated by isoprenaline (Figure 6 A, B, D).

### Combinations of Fluorescent Proteins for Coexpression Analyses

To ensure combinations of the seven fluorescent proteins could be used for coexpression analyses, OP 6 cells were transiently transfected with individual β_2_AR fusions and subsets that were not excited at overlapping wavelengths were mixed together. The following mixes were made [β_2_AR::Cerulean, β_2_AR::Venus, β_2_AR::mCherry], [β_2_AR::Teal, β_2_AR::Venus, β_2_AR::mCherry], and [β_2_AR::GFP, β_2_AR::mCherry, β_2_AR::AFP].

**Figure 7:**
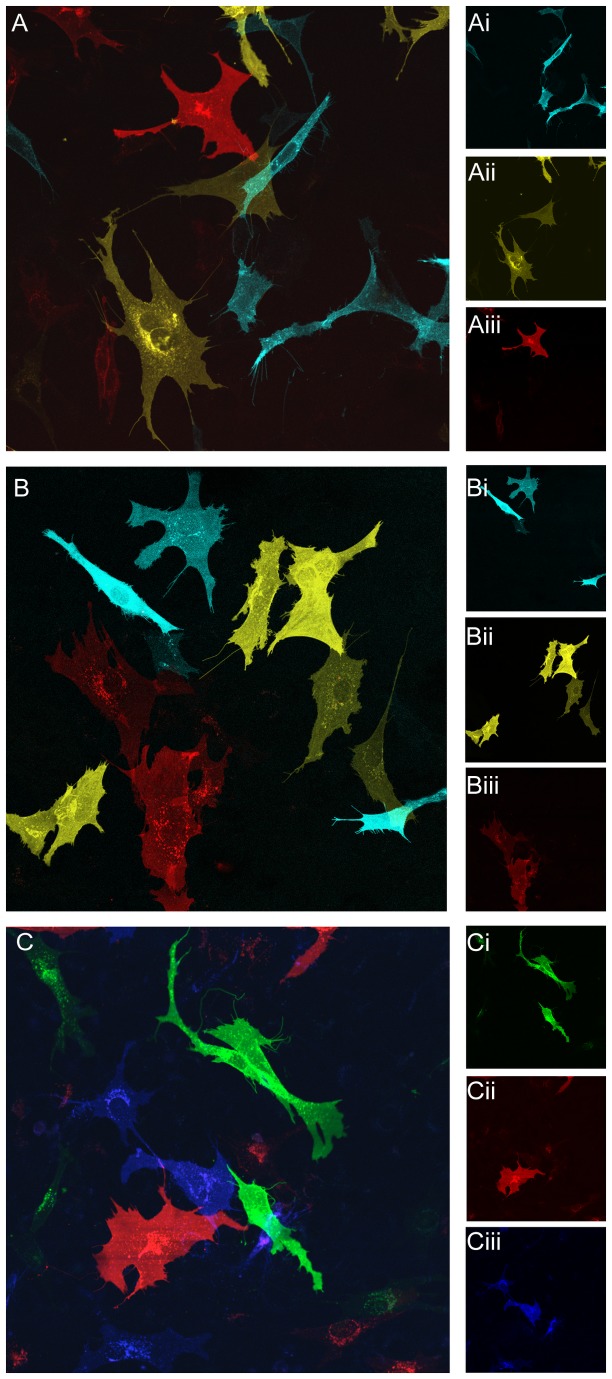
Visualization of OP 6 cells transiently expressing individual β_2_AR::XFPs and mixed. Mixed OP 6 cells expressing [β_2_AR::Cerulean, β_2_AR::Venus, β_2_AR::mCherry] in (A), [β_2_AR::Teal, β_2_AR::Venus, β_2_AR::mCherry] in (B) and [β_2_AR::GFP, β_2_AR::mCherry, β_2_AR::AFP] in (C). Each β_2_AR::XFP fusion was solely identified in its respective channel (Ai, Cerulean, Bi, Venus, Ci, mCherry), (Aii, Teal, Bii, Venus, Cii, mCherry), (Aiii, GFP, Biii, mCherry, Ciii, AFP).

The mixtures of [β_2_AR::Cerulean, β_2_AR::Venus, β_2_AR::mCherry] and [β_2_AR::Teal, β_2_AR::Venus, β_2_AR::mCherry] were excited at wavelengths 458, 512, and 561 respectively. No bleed-through was observed into any other channels (Figure 7 A, Ai-Aiii and B, Bi-Biii). The mixture of [β_2_AR::GFP, β_2_AR::mCherry, β_2_AR::AFP] was excited by wavelengths 488, 561, and 633, respectively. Bleed-through was only observed for AFP within perinuclear aggregates in the 561nm channel (Figure 7 C, Ci-Ciii).

## Discussion

Here, we have determined that some of the most useful fluorescent proteins do not affect the mouse β_2_AR trafficking and function when fused to its carboxyl terminus, consistent with the previous studies of human β_2_AR::GFP [11,12,13]. Since the β_2_AR is the archetype of GPCRs, it is likely that these fluorescent proteins do not affect trafficking and ligand response for other canonical GPCRs, providing a tool that makes the live visualization of protein localization, trafficking, and protein-protein interaction easier and efficient.

To determine the effects of XFPs on GPCRs, the ability of β_2_AR::XFPs were analyzed in the following ways: trafficking to the plasma membrane, internalization and trafficking to the early endosomes after ligand stimulation, regulation by β-Arrestin2 after ligand stimulation, and concentration dependent response to ligand. Our results confirm and extend previous findings. All of the β_2_AR::XFPs show functionality identical to that of β_2_AR::GFP whose behavior has been previously shown to be indistinguishable from β_2_ AR WT [11,12,13].

We show that our mouse β_2_AR::XFPs and the untagged mouse β_2_ AR WT have equivalent dose response profiles to that of human β_2_AR::GFP, which has been previously described. Importantly, the EC50 values, an indirect measure of the affinity of the receptor to ligand, were not affected and suggest a proper folding and integration of the fusion proteins into the cell membrane. All β_2_AR::XFPs showed lower maximum RFUs as compared to β_2_ AR WT. This result could be due to slightly less protein being trafficked to the plasma membrane or slight steric hindrance caused by the intracellular XFPs, which could reduce the transduction efficacy without affecting the ligand affinity.

Although each fluorescent protein did not affect β_2_AR function, there were notable differences between fluorescent proteins that could affect their application. Differences were within detectability, photostability, and aggregation within intracellular compartments. Venus was the best fluorescent protein, both untagged and as fusions to the gap peptide and β_2_AR. After Venus, in order of detection ease, were GFP, Teal, mCherry, mCerulean3, Cerulean, and AFP. This order remained true whether fused or unfused. mCerulean3, an improved version of Cerulean, was more easily detected with lower instances of photobleaching, as previously described. However, Teal was considerably easier to detect and the most photostable of the cyans, which is also consistent with the literature [26]. Cerulean was the most vulnerable to photobleaching. When fused to β_2_AR and the gap peptide, mCherry and AFP were the most susceptible to aggregation in subcellular compartments, likely due to cellular toxicity.

Our protein trafficking analyses made use of OP 6 cells (an olfactory neuronal precursor) [30], which vary in morphology, but all share the feature of many filopodia. When the untagged fluorescent proteins are expressed in OP 6 cells, they localize to the cytoplasm with few to no filopodia visible (Figure S2 Ai-Gi). However, when each fluorescent protein is tagged with the gap peptide, all of the gap::XFPs localize to the plasma membrane, and are expressed at the filopodia (Figure 1 Ai-Gi). Localization of untagged fluorescent proteins were rarely found in filopodia (~1 per OP6 cell) as compared to the gap tagged fluorescent proteins, which marked ~12 filopodia in nearly all OP 6 cells (Figure 2). This is consistent with the membrane association of the palmitoylated gap peptide, whose localization has been previously described in the filopodia of neuronal and non-neuronal cells [32,33].

Using this evidence, we have defined protein expression at the plasma membrane by its presence within filopodia. OP 6 cells are an efficient platform to measure plasma membrane trafficking efficacy of a protein. All seven β_2_AR fluorescent protein fusions were robustly found in the filopodia at ~14 filopodia in nearly all OP 6 cells. Thus, localization of the β_2_AR::XFPs were indistinguishable from that of gap::XFPs. In the future, OP 6 cells can be used to analyze the mechanisms of plasma membrane trafficking of β_2_AR::XFPs and other GPCR::XFPs.

A large number of GPCRs cannot be expressed in heterologous cell lines. This failure has prevented both structure-function and crystallographic analyses. Expression of mutant GPCRs::XFPs in OP 6 cells provides a high-throughput platform for analysis of plasma membrane trafficking using filopodia counts as an indicator of trafficking success. Moreover, OP 6 cells can be used to study the regulation of GPCRs during ligand activation, recycling after ligand activation, and G-protein coupling. Multiple fluorescent proteins can be coexpressed, providing visualization of subcellular localization of mutants in comparison to wild type receptors. This system is ideal for applications of live cell imaging, and utilizing time course analyses of subcellular processes, eliminating the need for antibodies in these applications.

Seven β_2_AR fluorescent protein fusions were constructed so that multiple versions could be studied simultaneously using different excitation wavelengths. GFP has been the most common choice for making fusion proteins *in vitro* and *in vivo*, however, its broad excitation spectra overlaps with that of Cerulean, mCerulean3, Teal and Venus. Therefore, coexpression analyses in this spectral range is limited to a cyan fluorescent protein and a yellow fluorescent protein, which do not have overlapping excitation. There are several red fluorescent proteins, with mCherry being the brightest monomer and having an excitation spectra the furthest shifted from Venus [28]. Our AFP (TagRFP657 T10K) is the brightest far-red monomer with the furthest excitation spectra shifted from mCherry [29]. In mixture analysis experiments we show that β_2_AR::Venus is readily distinguishable from both β_2_AR::Cerulean, β_2_AR::Teal and β_2_AR::mCherry (Figure 7 A, B). We also show that two red fluorescent protein fusions, β_2_AR::mCherry and β_2_AR::AFP, can be used in combination with β_2_AR::GFP (Figure 7 C) permitting the use of common GFP fusions in multiple coexpression analyses.

## Supporting Information

Figure S1 **Plasmid design for untagged fluorescent proteins, gap::XFPs, β_2_AR::XFPs.** The peGFP-N1 vector backbone provided by ClonTech was modified to remove the EcoRI- GFP- NotI coding sequence and insert an AscI site. (A) The untagged fluorescent proteins were cloned in with EcoRI or *EcoRI/NotI*. (B) gap::XFPs were cloned into this backbone with EcoRI. (C) For β_2_AR::XFPs the XFPs were cloned into a β_2_AR backbone with *PacI/NotI*.
(TIF)

Figure S2 **OP 6 cells transiently expressing untagged fluorescent proteins.** Single cells expressing each untagged fluorescent protein localizes in the cytoplasm (A-G). Expression does not extend to the filopodia (Ai-Gi magnified images).
(TIF)

Figure S3 **OP 6 cells do not have endogenous isoprenaline sensitivity or β_2_AR immunoreactivity.** Coexpression of gap::Teal and untagged Venus do not internalize upon isoprenaline exposure (A, 0 and 20 minutes post exposure). Antibody to β_2_AR reveals no endogenous activity in OP 6 cells transiently expressing gap::GFP (Bi, gap::GFP in green, Bii anti-β_2_AR in red, Biii overlap) as compared to cells transiently expressing unfused mouse β_2_ AR WT (C, anti-β_2_AR in red).
(TIF)

Figure S4 **Individual dose response curves for β_2_AR::XFPs.** Cells expressing each β_2_AR::XFP and human Gα15 were exposed to increasing concentrations of ligand isoprenaline and analyzed using the FLIPR assay. β_2_ AR WT has an EC50 value of 2.6 x 10^-9^. EC50 values for each fluorescent protein fusion, respectively, were as follows: (A) β_2_AR::GFP human, 3.1 x 10^-9^, (B) β_2_AR::GFP truncated, no EC50 (C) β_2_AR::Cerulean 4.0 x 10^-9^, (D) β_2_AR::mCerulean3 5.2 x 10^-9^, (E) β_2_AR::Teal 3.0 x 10^-9^, (F) β_2_AR::GFP 4.7 x 10^-9^, (G) β_2_AR::Venus 2.9 x 10^-9^, (H) β_2_AR::mCherry 4.0 x 10^-9^, and (I) β_2_AR::AFP 2.7 x 10^-9^.
(TIF)

File S1 **Nucleotide and amino acid sequences used to make untagged XFPs, gap::XFPs and β_2_AR::XFPs.**
(DOCX)

Movie S1 **Live imaging of β_2_AR::Cerulean rapid internalization after exposure to isoprenaline.** A single cell expressing β_2_AR::Cerulean exhibits diffuse expression at the plasma membrane. After 20 minutes of isoprenaline exposure, β_2_AR::Cerulean becomes punctate and internalized, with some protein remaining at the plasma membrane. This one-hour time course for β_2_AR::Cerulean reveals dynamic remodeling of the cell.
(MOV)

Movie S2 **Live imaging of β_2_AR::mCerulean3 rapid internalization after exposure to isoprenaline.** A single cell expressing β_2_AR::mCerulean3 exhibits diffuse expression at the plasma membrane. Twenty minute time-lapse imaging after isoprenaline exposure shows β_2_AR::mCerulean3 becoming punctate and internalized, with some protein remaining at the plasma membrane.
(MOV)

Movie S3 **Live imaging of β_2_AR::Teal rapid internalization after exposure to isoprenaline.** A single cell expressing β_2_AR::Teal exhibits diffuse expression at the plasma membrane. Twenty minute time-lapse imaging after isoprenaline exposure shows β_2_AR::Teal becoming punctate and internalized, with some protein remaining at the plasma membrane.
(MOV)

Movie S4 **Live imaging of β_2_AR::GFP rapid internalization after exposure to isoprenaline.** A single cell expressing β_2_AR::GFP exhibits diffuse expression at the plasma membrane. Twenty minute time-lapse imaging after isoprenaline exposure shows β_2_AR::GFP becoming punctate and internalized, with some protein remaining at the plasma membrane.
(MOV)

Movie S5 **Live imaging of β_2_AR::Venus rapid internalization after exposure to isoprenaline.** A single cell expressing β_2_AR::Venus exhibits diffuse expression at the plasma membrane. Twenty minute time-lapse imaging after isoprenaline exposure shows β_2_AR::Venus becoming punctate and internalized, with some protein remaining at the plasma membrane.
(MOV)

Movie S6 **Live imaging of β_2_AR::mCherry rapid internalization after exposure to isoprenaline.** A single cell expressing β_2_AR::mCherry exhibits diffuse expression at the plasma membrane. Twenty minute time-lapse imaging after isoprenaline exposure shows β_2_AR::mCherry becoming punctate and internalized, with some protein remaining at the plasma membrane.
(MOV)

Movie S7 **Live imaging of β_2_AR::AFP rapid internalization after exposure to isoprenaline.** A single cell expressing β_2_AR::AFP exhibits diffuse expression at the plasma membrane. Twenty minute time-lapse imaging after isoprenaline exposure shows β_2_AR::AFP becoming punctate and internalized, with some protein remaining at the plasma membrane.
(MOV)

Movie S8 **Live imaging of β_2_AR::GFP shows no internalization in the absence of isoprenaline.** A single cell expressing β_2_AR::GFP exhibits diffuse expression at the plasma membrane. β_2_AR::GFP remains in the membrane and is not internalized after 20 minute time-lapse imaging in the absence of isoprenaline.
(MOV)
