# Supplementary figures and images for: β2 Adrenergic Receptor Fluorescent Protein Fusions Traffic to the Plasma Membrane and Retain Functionality
Source: PLoS One. 2013 Sep 23;8(9):e74941. doi: 10.1371/journal.pone.0074941 (PMC3781101; doi:10.1371/journal.pone.0074941)

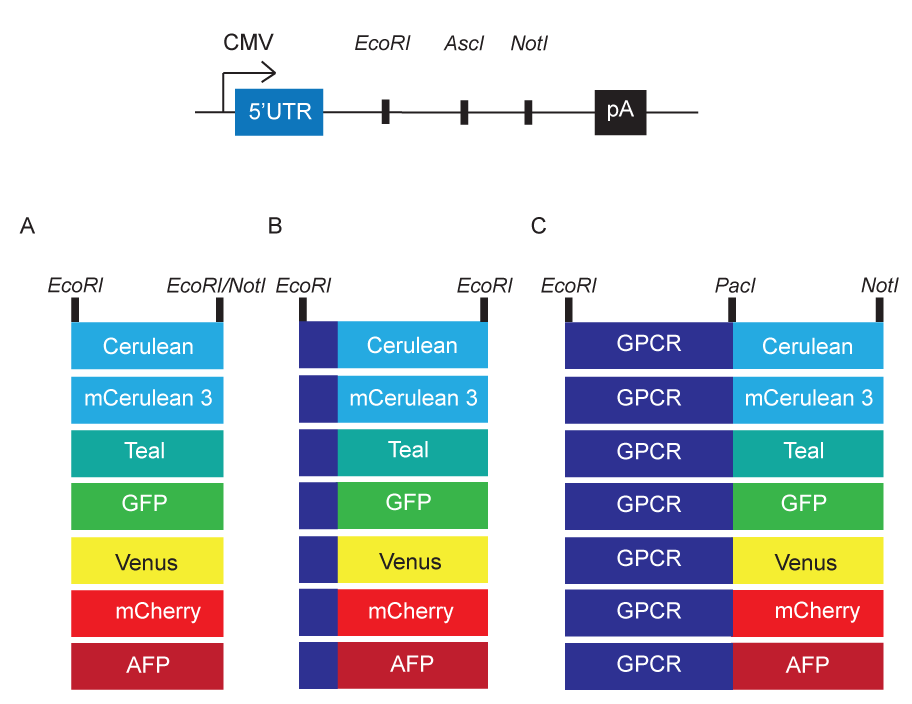

Supplement: Figure S1 — Plasmid design for untagged fluorescent proteins, gap::XFPs, β2AR::XFPs. The peGFP-N1 vector backbone provided by ClonTech was modified to remove the EcoRI- GFP- NotI coding sequence and insert an AscI site. (A) The untagged fluorescent proteins were cloned in with EcoRI or EcoRI/NotI. (B) gap::XFPs were cloned into this backbone with EcoRI. (C) For β2AR::XFPs the XFPs were cloned into a β2AR backbone with PacI/NotI. (TIF) [file pone.0074941.s001.tif]

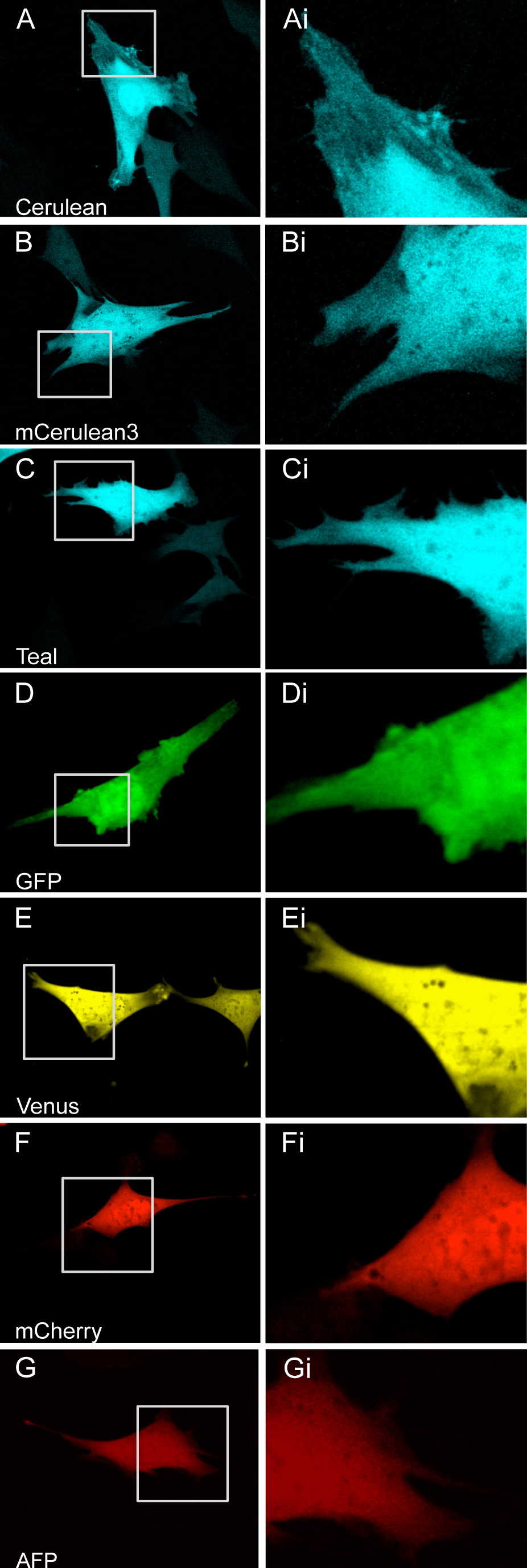

Supplement: Figure S2 — OP 6 cells transiently expressing untagged fluorescent proteins. Single cells expressing each untagged fluorescent protein localizes in the cytoplasm (A-G). Expression does not extend to the filopodia (Ai-Gi magnified images). (TIF) [file pone.0074941.s002.tif]

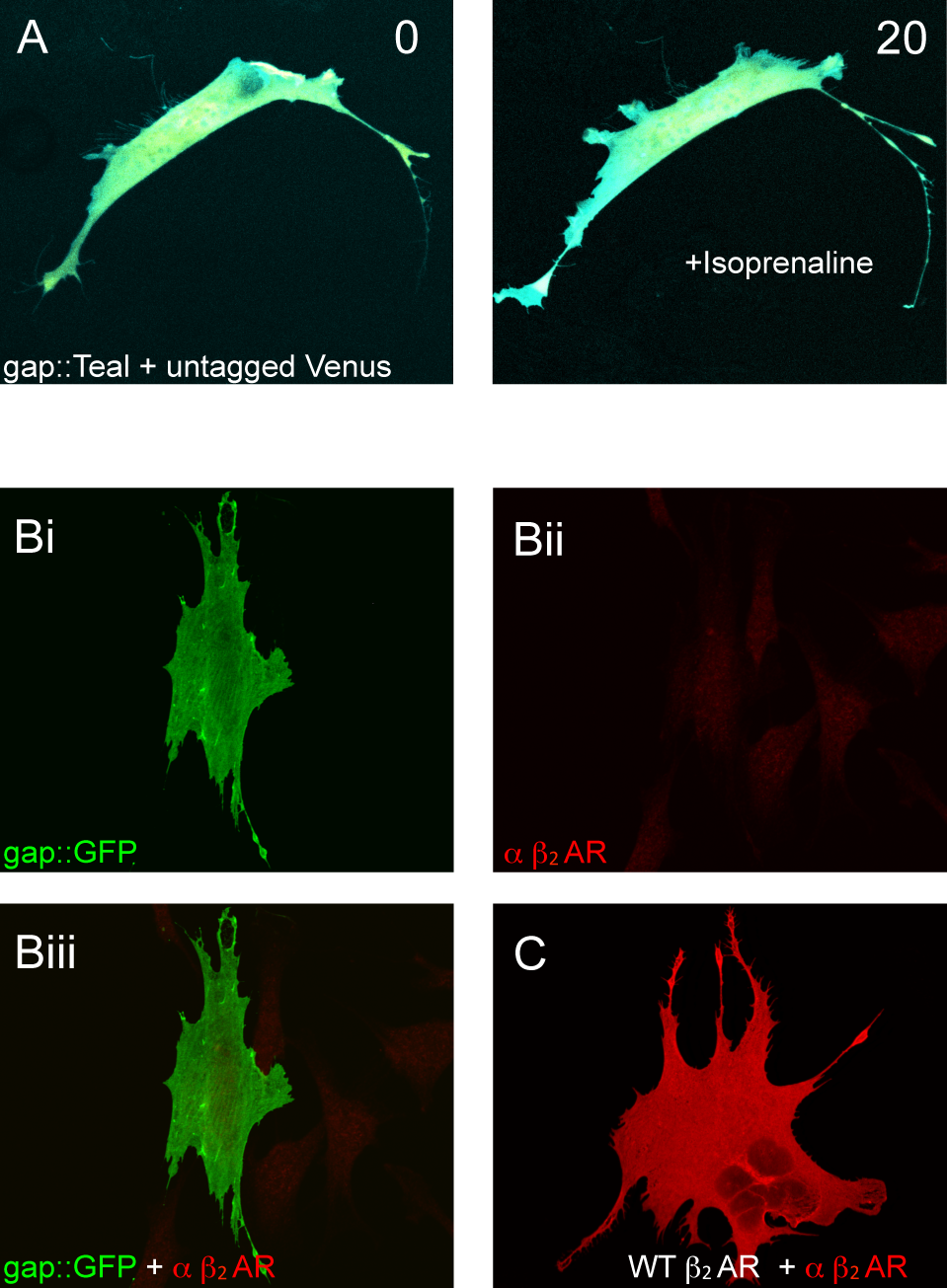

Supplement: Figure S3 — OP 6 cells do not have endogenous isoprenaline sensitivity or β2AR immunoreactivity. Coexpression of gap::Teal and untagged Venus do not internalize upon isoprenaline exposure (A, 0 and 20 minutes post exposure). Antibody to β2AR reveals no endogenous activity in OP 6 cells transiently expressing gap::GFP (Bi, gap::GFP in green, Bii anti-β2AR in red, Biii overlap) as compared to cells transiently expressing unfused mouse β2 AR WT (C, anti-β2AR in red). (TIF) [file pone.0074941.s003.tif]

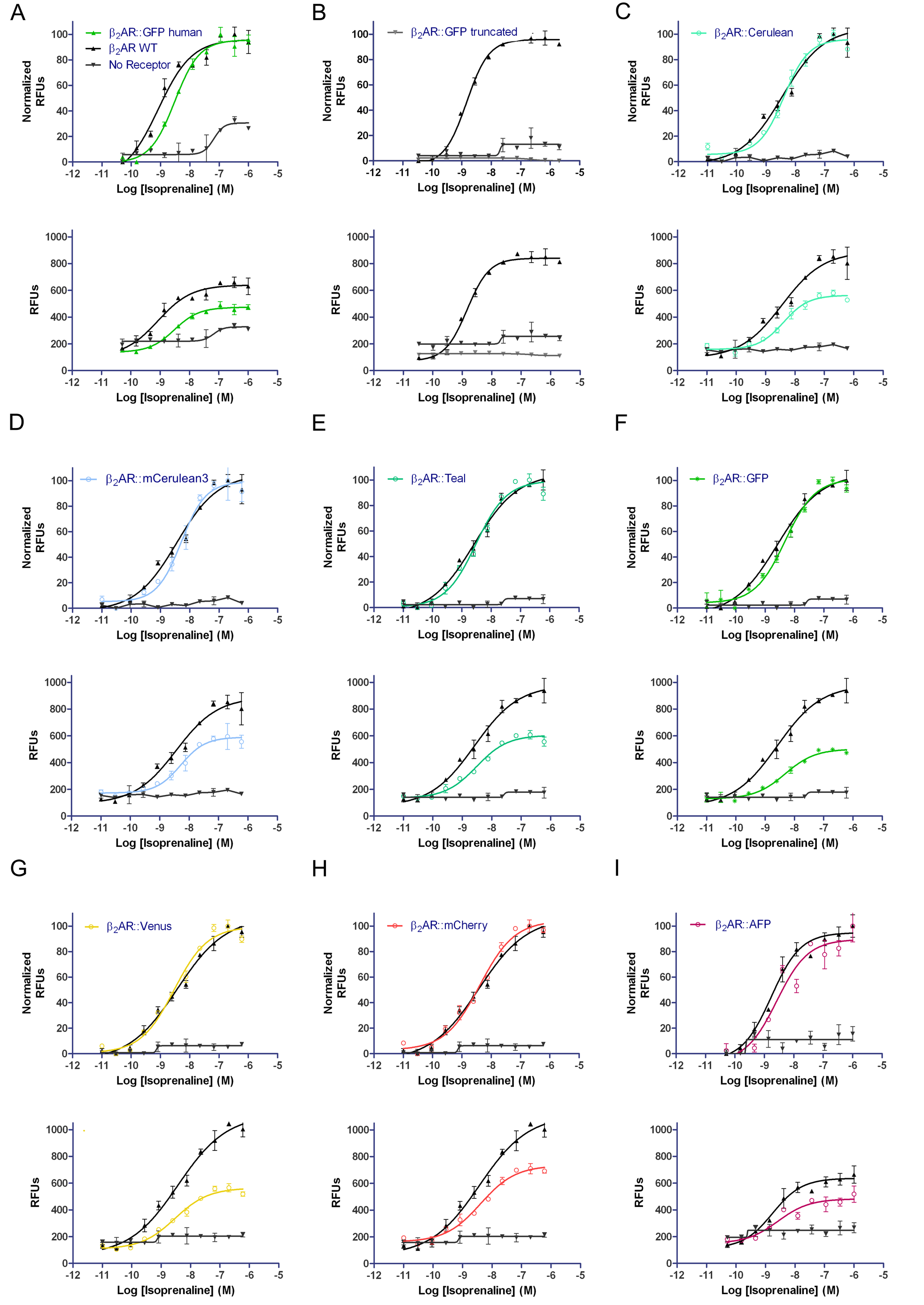

Supplement: Figure S4 — Individual dose response curves for β2AR::XFPs. Cells expressing each β2AR::XFP and human Gα15 were exposed to increasing concentrations of ligand isoprenaline and analyzed using the FLIPR assay. β2 AR WT has an EC50 value of 2.6 x 10^-9^. EC50 values for each fluorescent protein fusion, respectively, were as follows: (A) β2AR::GFP human, 3.1 x 10^-9^, (B) β2AR::GFP truncated, no EC50 (C) β2AR::Cerulean 4.0 x 10^-9^, (D) β2AR::mCerulean3 5.2 x 10^-9^, (E) β2AR::Teal 3.0 x 10^-9^, (F) β2AR::GFP 4.7 x 10^-9^, (G) β2AR::Venus 2.9 x 10^-9^, (H) β2AR::mCherry 4.0 x 10^-9^, and (I) β2AR::AFP 2.7 x 10^-9^. (TIF) [file pone.0074941.s004.tif]
