# Supplementary material for: β2 Adrenergic Receptor Fluorescent Protein Fusions Traffic to the Plasma Membrane and Retain Functionality
Source: PLoS One. 2013 Sep 23;8(9):e74941. doi: 10.1371/journal.pone.0074941 (PMC3781101; doi:10.1371/journal.pone.0074941)
Supplement: File S1 — Nucleotide and amino acid sequences used to make untagged XFPs, gap::XFPs and β2AR::XFPs. (DOCX) [file pone.0074941.s005.docx]

*Highlighted regions in yellow represent GFP amino and carboxy sequences*

**Cerulean:**

Amino acid sequence:

MVSKGEELFTGVVPILVELDGDVNGHKFSVSGEGEGDATYGKLTLKFICTTGKLPVPWPTLVTTLTWGVQCFARYPDHMKQHDFFKSAMPEGYVQERTIFFKDDGNYKTRAEVKFEGDTLVNRIELKGIDFKEDGNILGHKLEYNAISDNVYITADKQKNGIKANFKIRHNIEDGSVQLADHYQQNTPIGDGPVLLPDNHYLSTQSALSKDPNEKRDHMVLLEFVTAAGITLGMDELYK

Nucleotide sequence:

atggtgagcaagggcgaggagctgttcaccggggtggtgcccatcctggtcgagctggacggcgacgtaaacggccacaagttcagcgtgtccggcgagggcgagggcgatgccacctacggcaagctgaccctgaagttcatctgcaccaccggcaagctgcccgtgccctggcccaccctcgtgaccaccctgacctggggcgtgcagtgcttcgcccgctaccccgaccacatgaagcagcacgacttcttcaagtccgccatgcccgaaggctacgtccaggagcgcaccatcttcttcaaggacgacggcaactacaagacccgcgccgaggtgaagttcgagggcgacaccctggtgaaccgcatcgagctgaagggcatcgacttcaaggaggacggcaacatcctggggcacaagctggagtacaacgccatcagcgacaacgtctatatcaccgccgacaagcagaagaacggcatcaaggccaacttcaagatccgccacaacatcgaggacggcagcgtgcagctcgccgaccactaccagcagaacacccccatcggcgacggccccgtgctgctgcccgacaaccactacctgagcacccagtccgccctgagcaaagaccccaacgagaagcgcgatcacatggtcctgctggagttcgtgaccgccgccgggatcactctcggcatggacgagctgtacaagtaa

**mCerulean3:**

Amino acid sequence:

MVSKGEELFTGVVPILVELDGDVNGHKFSVSGEGEGDATYGKLTLKFICTTGKLPVPWPTLVTTLSWGVQCFARYPDHMKQHDFFKSAMPEGYVQERTIFFKDDGNYKTRAEVKFEGDTLVNRIELKGIDFKEDGNILGHKLEYNAIHGNVYITADKQKNGIKANFGLNCNIEDGSVQLADHYQQNTPIGDGPVLLPDNHYLSTQSKLSKDPNEKRDHMVLLEFVTAAGITLGMDELYK

Nucleotide sequence:

atggtgagcaagggcgaggagctgttcaccggcgtggtgcctatcctggtggagctggacggcgacgtgaacggccacaagttcagcgtgagcggcgagggcgagggcgacgccacctacggcaagctgaccctgaagttcatctgcaccaccggcaagctgcctgtgccttggcctacattggtcacaacattatcttggggcgtgcagtgcttcgccagataccctgaccacatgaagcagcacgacttcttcaagagcgccatgcctgagggctacgtgcaggagaggaccatcttcttcaaggacgacggcaactacaagaccagggccgaggtgaagttcgagggcgacaccctggtgaacaggatcgagctgaagggcatcgacttcaaggaggacggcaacatcctgggccacaagctggagtacaacgccatccacggcaacgtgtacatcaccgccgacaagcagaagaacggcatcaaggccaacttcggcctgaactgcaacatcgaggacggcagcgtgcagctggccgaccactaccagcagaacacccctatcggcgacggccctgtgctgctgcctgacaaccactacttatctactcaaagtaaattgtccaaggaccctaacgagaagagggaccacatggtgctgctggagttcgtgaccgccgccggcatcaccctgggcatggacgagctgtacaagtaa

**Teal:**

Amino acid sequence:

MVSKGEETTMGVIKPDMKIKLKMEGNVNGHAFVIEGEGEGKPYDGTNTINLEVKEGAPLPFSYDILTTAFAYGNRAFTKYPDDIPNYFKQSFPEGYSWERTMTFEDKGIVKVKSDISMEEDSFIYEIHLKGENFPPNGPVMQKKTTGWDASTERMYVRDGVLKGDVKHKLLLEGGGHHRVDFKTIYRAKKAVKLPDYHFVDHRIEILNHDKDYNKVTVYESAVARNSTDGMDELYK

Nucleotide sequence:

atggtgagcaagggcgaggagaccacaatgggcgtaatcaagcccgacatgaagatcaagctgaagatggagggcaacgtgaatggccacgccttcgtgatcgagggcgagggcgagggcaagccctacgacggcaccaacaccatcaacctggaggtgaaggagggagcccccctgcccttctcctacgacattctgaccaccgcgttcgcctacggcaacagggccttcaccaagtaccccgacgacatccccaactacttcaagcagtccttccccgagggctactcttgggagcgcaccatgaccttcgaggacaagggcatcgtgaaggtgaagtccgacatctccatggaggaggactccttcatctacgagatacacctcaagggcgagaacttcccccccaacggccccgtgatgcagaagaagaccaccggctgggacgcctccaccgagaggatgtacgtgcgcgacggcgtgctgaagggcgacgtcaagcacaagctgctgctggagggcggcggccaccaccgcgttgacttcaagaccatctacagggccaagaaggcggtgaagctgcccgactatcactttgtggaccaccgcatcgagatcctgaaccacgacaaggactacaacaaggtgaccgtttacgagagcgccgtggcccgcaactccaccgacggcatggacgagctgtacaagtaa

**GFP:**

Amino acid sequence:

MVSKGEELFTGVVPILVELDGDVNGHKFSVSGEGEGDATYGKLTLKFICTTGKLPVPWPTLVTTLTYGVQCFSRYPDHMKQHDFFKSAMPEGYVQERTIFFKDDGNYKTRAEVKFEGDTLVNRIELKGIDFKEDGNILGHKLEYNYNSHNVYIMADKQKNGIKVNFKIRHNIEDGSVQLADHYQQNTPIGDGPVLLPDNHYLSTQSALSKDPNEKRDHMVLLEFVTAAGITLGMDELYK

Nucleotide sequence:

atggtgagcaagggcgaggagctgttcaccggggtggtgcccatcctggtcgagctggacggcgacgtaaacggccacaagttcagcgtgtccggcgagggcgagggcgatgccacctacggcaagctgaccctgaagttcatctgcaccaccggcaagctgcccgtgccctggcccaccctcgtgaccaccctgacctacggcgtgcagtgcttcagccgctaccccgaccacatgaagcagcacgacttcttcaagtccgccatgcccgaaggctacgtccaggagcgcaccatcttcttcaaggacgacggcaactacaagacccgcgccgaggtgaagttcgagggcgacaccctggtgaaccgcatcgagctgaagggcatcgacttcaaggaggacggcaacatcctggggcacaagctggagtacaactacaacagccacaacgtctatatcatggccgacaagcagaagaacggcatcaaggtgaacttcaagatccgccacaacatcgaggacggcagcgtgcagctcgccgaccactaccagcagaacacccccatcggcgacggccccgtgctgctgcccgacaaccactacctgagcacccagtccgccctgagcaaagaccccaacgagaagcgcgatcacatggtcctgctggagttcgtgaccgccgccgggatcactctcggcatggacgagctgtacaagtaa

**Venus:**

Amino Acid Sequence:

MVSKGEELFTGVVPILVELDGDVNGHKFSVSGEGEGDATYGKLTLKLICTTGKLPVPWPTLVTTLGYGLQCFARYPDHMKQHDFFKSAMPEGYVQERTIFFKDDGNYKTRAEVKFEGDTLVNRIELKGIDFKEDGNILGHKLEYNYNSHNVYITADKQKNGIKANFKIRHNIEDGGVQLADHYQQNTPIGDGPVLLPDNHYLSYQSALSKDPNEKRDHMVLLEFVTAAGITLGMDELYK

Nucleotide Sequence:

atggtgagcaagggcgaggagctgttcaccggggtggtgcccatcctggtcgagctggacggcgacgtaaacggccacaagttcagcgtgtccggcgagggcgagggcgatgccacctacggcaagctgaccctgaagctgatctgcaccaccggcaagctgcccgtgccctggcccaccctcgtgaccaccctgggctacggcctgcagtgcttcgcccgctaccccgaccacatgaagcagcacgacttcttcaagtccgccatgcccgaaggctacgtccaggagcgcaccatcttcttcaaggacgacggcaactacaagacccgcgccgaggtgaagttcgagggcgacaccctggtgaaccgcatcgagctgaagggcatcgacttcaaggaggacggcaacatcctggggcacaagctggagtacaactacaacagccacaacgtctatatcaccgccgacaagcagaagaacggcatcaaggccaacttcaagatccgccacaacatcgaggacggcggcgtgcagctcgccgaccactaccagcagaacacccccatcggcgacggccccgtgctgctgcccgacaaccactacctgagctaccagtccgccctgagcaaagaccccaacgagaagcgcgatcacatggtcctgctggagttcgtgaccgccgccgggatcactctcggcatggacgagctgtacaagtaa

**mCherry:**

Amino Acid Sequence:

MVSKGEEDNMAIIKEFMRFKVHMEGSVNGHEFEIEGEGEGRPYEGTQTAKLKVTKGGPLPFAWDILSPQFMYGSKAYVKHPADIPDYLKLSFPEGFKWERVMNFEDGGVVTVTQDSSLQDGEFIYKVKLRGTNFPSDGPVMQKKTMGWEASSERMYPEDGALKGEIKQRLKLKDGGHYDAEVKTTYKAKKPVQLPGAYNVNIKLDITSHNEDYTIVEQYERAEGRHSTGGMDELYK

Nucleotide Sequence:

atggtgagcaagggcgaggaggataacatggccatcatcaaggagttcatgcgcttcaaggtgcacatggagggctccgtgaacggccacgagttcgagatcgagggcgagggcgagggccgcccctacgagggcacccagaccgccaagctgaaggtgaccaagggtggccccctgcccttcgcctgggacatcctgtcccctcagttcatgtacggctccaaggcctacgtgaagcaccccgccgacatccccgactacttgaagctgtccttccccgagggcttcaagtgggagcgcgtgatgaacttcgaggacggcggcgtggtgaccgtgacccaggactcctccctgcaggacggcgagttcatctacaaggtgaagctgcgcggcaccaacttcccctccgacggccccgtaatgcagaagaagaccatgggctgggaggcctcctccgagcggatgtaccccgaggacggcgccctgaagggcgagatcaagcagaggctgaagctgaaggacggcggccactacgacgctgaggtcaagaccacctacaaggccaagaagcccgtgcagctgcccggcgcctacaacgtcaacatcaagttggacatcacctcccacaacgaggactacaccatcgtggaacagtacgaacgcgccgagggccgccactccaccggcggcatggacgagctgtacaagtaa

**AFP (TagRFP657 T10K):**

MVSKGEELIKENMHMKLYMEGTVNNHHFKCTSEGEGKPYEGTQTQRIKVVEGGPLPFAFDILATSFMYGSHTFINHTQGIPDFWKQSFPEGFTWERVTTYEDGGVLTATQDTSLQDGCLIYNVKIRGVNFPSNGPVMQKKTLGWEAHTEMLYPADGGLEGRTALALKLVGGGHLICNFKTTYRSKKPAKNLKMPGVYYVDYRLERIKEADKETYVEQHEVAVARYCDLPSKLGHKLNGMDELYK

atggtgagcaagggcgaggagctgatcaaggagaacatgcacatgaagctgtacatggagggcaccgtgaacaaccaccacttcaagtgcaccagcgagggcgagggcaagccctacgagggcacccagacccagagaatcaaggtggtggagggcggccccctgcccttcgccttcgacatcctggccaccagcttcatgtacggcagccacaccttcatcaaccacacccagggcatccccgacttctggaagcagagcttccccgagggcttcacctgggagagagtgaccacctacgaggacggcggcgtgctgaccgccacccaggacaccagcctgcaggacggctgcctgatctacaacgtgaagatcagaggcgtgaacttccccagcaacggccccgtgatgcagaagaagaccctgggctgggaggcccacaccgagatgctgtaccccgccgacggcggcctggagggcagaaccgccctggccctgaagctggtgggcggcggccacctgatctgcaacttcaagaccacctacagaagcaagaagcccgccaagaacctgaagatgcccggcgtgtactacgtggactacagactggagagaatcaaggaggccgacaaggagacctacgtggagcagcacgaggtggccgtggccagatactgcgacctgcccagcaagctgggccacaagctgaacggcatggacgagctgtacaagtga

**Gap tag for fusions:**

Amino Acid Sequence:

MLCCIRRTKPVEKNEEADQE

Nucleotide Sequence:

atgctgtgctgcatcagaagaactaagccggttgagaagaatgaagaggccgatcaggag

**Mouse β_2_AR for fusions:**

*Green highlight represents last amino acid in β_2_AR::GFP truncation*

Amino acid sequence:

MGPHGNDSDFLLAPNGSRAPDHDVTQERDEAWVVGMAILMSVIVLAIVFGNVLVITAIAKFERLQTVTNYFIISLACADLVMGLAVVPFGASHILMKMWNFGNFWCEFWTSIDVLCVTASIETLCVIAVDRYVAITSPFKYQSLLTKNKARVVILMVWIVSGLTSFLPIQMHWYRATHKKAIDCYTEETCCDFFTNQAYAIASSIVSFYVPLVVMVFVYSRVFQVAKRQLQKIDKSEGRFHAQNLSQVEQDGRSGHGLRRSSKFCLKEHKALKTLGIIMGTFTLCWLPFFIVNIVHVIRDNLIPKEVYILLNWLGYVNSAFNPLIYCRSPDFRIAFQELLCLRRSSSKTYGNGYSSNSNGRTDYTGEPNTCQLGQEREQELLCEDPPGMEGFVNCQGTVPSLSVDSQGRNCSTNDSPL

Nucleotide sequence:

atggggccacacgggaacgacagcgacttcttgctggcacccaacggaagccgagcgccagaccacgacgtcactcaggaacgggacgaagcgtgggttgtgggcatggccatcctcatgtcggttatcgtcctggccatcgtgtttggcaacgtgctggtcatcacggccattgccaagttcgagcgactacaaaccgtcaccaactacttcataatctccttggcgtgtgctgatctagtcatgggcctagcggtggtgccgtttggggccagtcacatccttatgaaaatgtggaattttggcaacttctggtgcgagttctggacttccattgatgtgttgtgcgtcacagccagcatcgagaccctgtgcgtgattgcagtggatcgctatgttgctatcacatcgcccttcaagtaccagagcctgctgaccaagaataaggcccgagtggtcatcctgatggtatggattgtatctggccttacctcctttttgcctatccagatgcactggtaccgtgccacccacaagaaagctatcgattgttacaccgaggagacttgctgtgacttcttcacgaaccaggcctacgccatcgcgtcctcgattgtgtctttctacgtgcccctggtggtgatggtctttgtctattcccgggtcttccaggtggccaaaaggcagctgcagaagatagacaaatctgaaggaagattccacgcccaaaacctcagccaggtggagcaggatgggcggagcggccacggactccgaaggtcctccaagttctgcttgaaagagcacaaagccctcaagactttaggcatcatcatgggcacattcaccctctgctggctgcccttcttcattgtcaatatcgtgcacgttatcagggacaacctcatccctaaggaagtttacattctccttaactggttgggctacgtcaactctgccttcaatcctcttatctactgtcggagtccagatttcaggattgcctttcaagagcttctgtgccttcgcaggtcttcttcgaaaacctatgggaacggctactctagcaatagcaacggcagaacggactacacaggggagccaaacacttgtcagctggggcaggagagagaacaggaactgctgtgtgaggatcccccaggcatggaaggctttgtgaactgtcaaggtactgtgcctagccttagcgttgactcccaaggaaggaactgtagtacaaatgactcgccactg

**Human β_2_AR for fusions:**

Amino Acid Sequence:

MGQPGNGSAFLLAPNRSHAPDHDVTQQRDEVWVVGMGIVMSLIVLAIVFGNVLVITAIAKFERLQTVTNYFITSLACADLVMGLAVVPFGAAHILMKMWTFGNFWCEFWTSIDVLCVTASIETLCVIAVDRYFAITSPFKYQSLLTKNKARVIILMVWIVSGLTSFLPIQMHWYRATHQEAINCYANETCCDFFTNQAYAIASSIVSFYVPLVIMVFVYSRVFQEAKRQLQKIDKSEGRFHVQNLSQVEQDGRTGHGLRRSSKFCLKEHKALKTLGIIMGTFTLCWLPFFIVNIVHVIQDNLIRKEVYILLNWIGYVNSGFNPLIYCRSPDFRIAFQELLCLRRSSLKAYGNGYSSNGNTGEQSGYHVEQEKENKLLCEDLPGTEDFVGHQGTVPSDNIDSQGRNCSTNDSLL

Nucleotide Sequence:

atggggcaacccgggaacggcagcgccttcttgctggcacccaatagaagccatgcgccggaccacgacgtcacgcagcaaagggacgaggtgtgggtggtgggcatgggcatcgtcatgtctctcatcgtcctggccatcgtgtttggcaatgtgctggtcatcacagccattgccaagttcgagcgtctgcagacggtcaccaactacttcatcacttcactggcctgtgctgatctggtcatgggcctggcagtggtgccctttggggccgcccatattcttatgaaaatgtggacttttggcaacttctggtgcgagttttggacttccattgatgtgctgtgcgtcacggccagcattgagaccctgtgcgtgatcgcagtggatcgctactttgccattacttcacctttcaagtaccagagcctgctgaccaagaataaggcccgggtgatcattctgatggtgtggattgtgtcaggccttacctccttcttgcccattcagatgcactggtaccgggccacccaccaggaagccatcaactgctatgccaatgagacctgctgtgacttcttcacgaaccaagcctatgccattgcctcttccatcgtgtccttctacgttcccctggtgatcatggtcttcgtctactccagggtctttcaggaggccaaaaggcagctccagaagattgacaaatctgagggccgcttccatgtccagaaccttagccaggtggagcaggatgggcggacggggcatggactccgcagatcttccaagttctgcttgaaggagcacaaagccctcaagacgttaggcatcatcatgggcactttcaccctctgctggctgcccttcttcatcgttaacattgtgcatgtgatccaggataacctcatccgtaaggaagtttacatcctcctaaattggataggctatgtcaattctggtttcaatccccttatctactgccggagcccagatttcaggattgccttccaggagcttctgtgcctgcgcaggtcttctttgaaggcctatgggaatggctactccagcaacggcaacacaggggagcagagtggatatcacgtggaacaggagaaagaaaataaactgctgtgtgaagacctcccaggcacggaagactttgtgggccatcaaggtactgtgcctagcgataacattgattcacaagggaggaattgtagtacaaatgactcactgctg

**β_2_AR linker for fusions:**

Amino acid sequence:

LINDPPVAT

Nucleotide sequence:

ttaattaacgatccaccggtcgccacc

**List of Clone Names:**

D346: Parent fusion vector

D356: β_2_AR::GFP Human

D357: β_2_AR::GFP

D858: β_2_AR::Teal

D859: β_2_AR::Venus

D860: β_2_AR::mCherry

D1188: β_2_AR::GFP Truncated

D1317: β_2_AR::Cerulean

D1328: β_2_AR::mCerulean3

D1501-4: β_2_AR::AFP

D1381: gap::GFP

D396: gap::Teal

D397: gap::Venus

D359: gap::mCherry

D1525-1: gap::AFP

D1193: gap::Cerulean

D1565-3: gap::mCerulean3

D1388-2: untagged GFP

D1518: untagged Teal

D1519: untagged Cerulean

D1520: untagged mCerulean3

D1521: untagged Venus

D1522: untagged mCherry

D1568-1: untagged AFP
